# Virtual screening and activity evaluation of multitargeting inhibitors for idiopathic pulmonary fibrosis

**DOI:** 10.3389/fphar.2022.998245

**Published:** 2022-09-08

**Authors:** Rui Wang, Jian Xu, Rong Yan, Huanbin Liu, Jingxin Zhao, Yuan Xie, Wenbin Deng, Weiping Liao, Yichu Nie

**Affiliations:** ^1^ Clinical Research Institute, The First People’s Hospital of Foshan, Foshan, China; ^2^ School of Pharmaceutical Sciences (Shenzhen), Shenzhen Campus of Sun Yat-sen University, Shenzhen, China; ^3^ Foshan Fourth People’s Hospital, Foshan, China

**Keywords:** idiopathic pulmonary fibrosis, virtual screening, machine learning, multitarget inhibitors, activity evaluation

## Abstract

Transforming growth factor β receptor (TGF-β1R) and receptor tyrosine kinases (RTKs), such as VEGFRs, PDGFRs and FGFRs are considered important therapeutic targets in blocking myofibroblast migration and activation of idiopathic pulmonary fibrosis (IPF). To screen and design innovative prodrug to simultaneously target these four classes of receptors, we proposed an approach based on network pharmacology combining virtual screening and machine learning activity prediction, followed by efficient *in vitro* and *in vivo* models to evaluate drug activity. We first constructed Collagen1A2-A549 cells with type I collagen as the main biomarker and evaluated the activity of compounds to inhibit collagen expression at the cellular level. The data from the first round of Collagen1A2-A549 cell screening were substituted into the machine learning model, and the model was optimized accordingly. As a result, the false positive rate of the model was reduced from 85.0% to 66.7%, and two prospective compounds, Z103080500 and Z104578368, were finally selected. Collagen levels were reduced effectively by both Z103080500 (67.88% reduction) and Z104578368 (69.54% reduction). Moreover, these two compounds showed low cellular cytotoxicity. Subsequently, the effect of Z103080500 and Z104578368 was evaluated in a bleomycin-induced C57BL/6 mouse IPF model. These results showed that 50 mg/kg Z103080500 and Z104578368 could effectively reduce the number of inflammatory cells and the expression level of α-SMA. Meanwhile, Z103080500 and Z104578368 reduced the expression of major markers and inflammatory factors of IPF, such as collagen, IFN-γ, IL-17 and HYP, indicating that these screened Z103080500 and Z104578368 effectively delayed lung tissue inflammation and had a potential therapeutic effect on IPF. Our findings demonstrate that a screening and evaluation model for prodrug against IPF has been successfully established. It is of great significance to further modify these compounds to enhance their potency and activity.

## Introduction

Idiopathic pulmonary fibrosis (IPF) is a chronic progressive disease with lung function decline. Some risk factors, such as senility, air pollution, and smoking, increase the prevalence of IPF (Thannickal et al., 2004; Snijder et al., 2019). The extracellular matrix produced by activated myofibroblasts in IPF patients leads to scarring and tissue stiffness, which ultimately results in loss of lung function. Transforming growth factor β receptor (TGF-β1R) and receptor tyrosine kinases (RTKs), such as VEGFRs, PDGFRs, and FGFRs, play important roles in the migration and activation of myofibroblasts (Mackinnon et al., 2012; MacKenzie et al., 2015; Biasin et al., 2020; Amano et al., 2021; Yang et al., 2021; Ye and Hu, 2021). The drug options for IPF treatment are limited. Currently, the drugs nintedanib (NDNB) and pirfenidone (PFD) have been used in the clinical treatment of IPF (King et al., 2014; Richeldi et al., 2014). NDNB is a tyrosine kinase inhibitor that selectively binds to VEGFRs, FGFRs, and PDGFRs and then inhibits the signals associated with proliferation, migration and transformation in fibroblasts (Richeldi et al., 2014; Guzy et al., 2017; Tepede and Yogaratnam, 2019). PFD, a potent cytokine inhibitor, exerts an antifibrotic effect by blocking the downstream signaling pathway of TGFβ1R (Taniguchi et al., 2010). However, NDNB and PFD only showed good clinical efficacy in some IPF patients. In addition, the side effects of these two drugs are numerous, including strong hepatorenal toxicity, high dose and drug resistance, which reduce patient compliance and limit their clinical application (Spagnolo et al., 2021). Therefore, novel low-toxicity small molecules were developed to simultaneously block TGF-β1R, VEGFRs, PDGFRs and FGFRs and meet the clinical treatment needs of IPF (Umemura et al., 2021).

Computer-aided drug design (CADD) is a new technology for research that has been developed in recent years (Iwasawa et al., 2019; Kaur and Khatik, 2021). The use of rational drug design, as applied in CADD, provides a knowledge-driven approach that can yield valuable information about the interaction patterns between proteins and ligands (complex), as well as the binding affinity. Furthermore, the availability of supercomputers, parallel processing, and advanced software have greatly facilitated the rate of lead identification in pharmaceutical research. This technology greatly shortens the time needed for new drug design and saves manpower and material resources for creating new drugs. In recent times, computer-aided drug design (CADD) strategies have been applied successfully in drug development processes. CADD includes receptor structure-based drug design (SBDD) and ligand-based drug design (LBDD) (Baig et al., 2018). For example, structure-based drug design strategies in the development of novel 5-LOX inhibitors have been reported (Aparoy et al., 2012). Based on the structure and properties of receptors, SBDD looks for ligand molecules that can specifically bind to the receptor, and virtual screening is an important method (Kaur and Khatik, 2021).

Here, we investigated novel lead compounds within clinically effective and selected representative compounds for CADD targeting of targets of IPF. For the positive candidates, modified cells were used to verify their activity for high-throughput screening. Finally, we evaluated the activity and cytotoxicity of the active agents in a bleomycin (BLM)-treated mouse model. The results identified two lead compounds that might be potential antifibrotic candidates.

## Materials and methods

### Correlation analysis of target sequence, spatial structure and physiology

The 3D structure and sequence of target proteins were obtained from the Protein Data Bank (https://www.rcsb.org/), which were VEGFR1 (PDB: 3Hng), VEGFR2 (PDB: 2OH4), and FGFR1 (PDB: 2Hng). 5A46), FGFR2 (PDB: 3RI1), FGFR3 (PDB: 4K33), PDGFRα (PDB: 5GRN), TGFβ1R (PDB: 3TZM). PyMol 2.1 software was used to remove elements that had no connection with targets from the model, such as water molecules and ligands. We performed structural overlap of the above targets and sequenced alignments. Then, structural overlap and sequence alignment of the above targets were carried out. Then, we analyzed the correlation of each target in the physiological pathway through the STRING database.

### Swiss-model homology simulation

Since there is no established model for VEGFR3 and PDGFRβ, we need to construct 3D models of VEGFR3 and PDGFRβ through homology simulation. Protein sequences of VEGFR3 (Identifier: p17948-1) and PDGFRβ (Identifier: p16234-1) were obtained from UniProt (https://www.UniProt.org/). Homologous simulation was completed with SWISS - MODEL (https://swissmodel.expasy.org/), in which the reference templates were VEGFR3 (Template: VEGFR2, PDB: 4 agc) and PDGFR beta (Template: FLT3, PDB: 4 rt7). RAMPGE (http://www-cryst.bioc.cam.ac.uk/rampage/) analysis was performed after establishing the models. Verify 3D was used using SAVESv6.0 (https://saves.mbi.ucla.edu/), drawing tools for Origin 9.0. We used PSIPRED PSIPRED 4.0 (http://bioinf.cs.ucl.ac.uk/psipred/), DISOPRED3, MEMSAT - SVM and pGen THREADER module disorder protein analysis. Subsequently, the String database (https://string-db.org/) was used to analyze the correlation of physiological effects of each target *in vivo*.

### Virtual screening of AutoDock

To ensure the diversity and efficiency of the database, we selected Diversity Libraries in Enamine (https://enamine.net/hit-finding) and removed the ligand database with a molecular weight of 370–960 (approximately 60,000 compounds). The Lamarckian genetic algorithm in AutoDock 4.2 was used for docking. Standard docking procedures are used for flexible ligands and rigid proteins. We used ADT tools to increase Koollman charges on ligands and proteins. At the same time, we set the Grid according to the existing ligands on the target so that the Grid can cover the whole ligand binding region. We calculated the binding affinities through a grid spacing of 0.6 A and the distance correlation function of permittivity. All other parameters are set by default. Finally, ADT was used to analyze the structure files of the compounds with the minimum binding energy.

### Machine learning model

Based on the known relationship between ligand structure and physiological activity, we constructed a machine learning model to predict the activity of candidate small molecules based on the resulting information. We obtained the structure files of the target’s existing ligands and the IC50 data from the DrugBank database (https://go.drugbank.com/) and Selleck database (at). We converted the ligand activity data into the −log⁡⁡(pIC50) form. Using the Python RDkit module MolecularDescriptorCalculator, procedures for existing ligands and screening of molecular characteristics of the database were extracted. StandardScaler of the Sklearn module was used to standardize the molecular characteristics. The combination feature selection based on random forest and RFE was carried out by using the Sklearn module. Finally, we used support vector machine (SVM), Adaboost (ADB), random forest (RF), and gradient boosting (GDB) in Python. The scikit-learn, K-nearest neighbor (KNN) and Bayesianridge (BR) algorithms were used to perform fitting calculations on the existing ligand data, and finally, a machine learning model for activity screening was obtained. Finally, the machine learning model was used to select the ligand with the highest activity value from the ligand database for analysis.

### Consensus scoring

According to the scoring rules set, compounds with the highest comprehensive score and potential were selected.

### Data feedback optimization model

After screening the reporter gene cells of collagen1A2-A549, the obtained compound expression data were fed back to the previous machine learning model for optimization of the machine learning model. The compounds with negative experimental results were substituted into the machine learning model as punishment items to obtain the optimized machine learning model. We hope to reduce the proportion of false positive compounds predicted by machine learning models.

### Constructed with modified cells

Primers were designed to clone the target promoter fragment of collagen1A2 from human genomic DNA by PCR, and the fragment was inserted into the luciferase reporter gene plasmid (pcDNATM3.1 VETORS, LandmBio ^®^, Guangzhou). Positive clones were screened and sequenced. The plasmid was amplified, cloned and purified for later use. Transcription factor plasmids were amplified and purified for later use. Meanwhile, the corresponding no-load plasmid control was prepared for purification and reserve. A549 cells were cultured and inoculated in 24-well plates for 24 h (80% confluence). G418 (50 mM) was used for screening. The reporter gene plasmid and transcription factor expression plasmid were cotransfected into cells. Collagen1A2-A549 cells were cultured and inoculated in 96-well plates for 12 h. PBS, TGFβ1 (10 ng/μL, 5 ng/μL and 1 ng/μL), FGF1 (10 ng/μL, 5 ng/μL and 1 ng/μL), PDGFα (10 ng/μL, 5 ng/μL and 1 ng/μL) (Biolegend ^®^, California) agonists were administered and cultured for 12, 24, and 36 h, respectively. PBS, PFD (10 μM, 1 μM and 0.5 μM), NDNB (10 μM, 1 μM, and 0.5 μM), and dexamethasone (10 μM, 1 μM and 0.5 μM) (Enamine, Ukraine) were cultured for 12, 24, and 36 h, respectively. After standard treatment using the Luciferase Reporter Gene Assay Kit (Yeasen^®^, Guangzhou), the assay was assayed at 570/10 nm using a multifunction Assay (PerkinElmer, Finland).

### Cytotoxicity test

A CCK-8 Cell Proliferation and Cytotoxicity Assay Kit (CK101-01, Data Invention Biotech Hong Kong, China) was used to test the cytotoxicity of the compounds. Collagen1A2-A549 cells with different concentrations were inoculated, and standard curves were obtained according to standard treatment. Collagen1A2-A549 cells were cultured in DMEM supplemented with 10% Foetal Bovine Serum (DIB-12B-10X50ML, Data Invention Biotech) and inoculated in 96-well plates for 24 h, and 6.25, 12.5, 25, 50, and 100 μM compounds and PBS were added. After incubation for 24 h, 10 μL of CCK8 reagent was added and incubated for 3 h. The absorbance value at 450 nm was determined by a microplate analyzer.

### Lead compounds were screened using modified Collagen1A2-A549 cells

Purchase 29 of these compounds from Enamine (https://enamine.net/hit-finding). Collagen1A2-A549 cells were cultured and inoculated in 96-well plates for 12 h. PBS and the compound were given 50 μM and cultured for 12 h, 24 h and 36 h, respectively. A Luciferase Reporter Gene Assay Kit (Yeasen^®^, Guangzhou) was used to assay at 570/10 nm.

### IPF mouse model induced by BLM

The Animal Center of Sun Yat-Sen University provided SPF male C57BL/6 mice (*n* = 70) at 6–8 weeks. Mice were fed adaptively in an SPF animal house for 1 week for the experiment. The mice were randomly divided into a blank group, model group, PFD group (M2328, Abmole, United States), NDNB group BIBF 1120, Abmole, United States, PFD + NDNB group, Z103080500 experimental group and Z104578368 group, with 10 mice in each group. On day 0, BLM (3 mg/kg, 40 μL) was injected into the trachea. On day 3, Z103080500 and Z104578368 were prepared into 50 mg/kg suspension, PFD was prepared into 100 mg/kg suspension, NDNB was prepared into 50 mg/kg suspension, NDNB 50 mg/kg + PFD 100 mg/kg suspension, and intragastric administration was performed daily.

### Weight and survival rate

To assess the survival of mice, body weight and mortality were recorded and observed periodically. On day 0, the mice were given 3 mg/kg BLM intragranically. The body weight of the mice and the survival rate of each group were recorded every 4 days.

### Histopathological observation of lung tissue

Lung tissues were fixed with 3.7% paraformaldehyde at 4 C overnight. Gradient ethanol was used to dehydrate the lungs, which were embedded into a wax block. A slicer (Leica, Germany) cuts slices 5–7 m thick. Slices were placed on polylysine-coated glass slides and kept at room temperature until further use. HE staining and Masson staining were performed according to standard procedures. The degree of lung fiber was evaluated according to the results of H&S staining according to the Ashcroft scoring standard. The blue part of the Masson’s trichrome-stained section was quantified using ImageJ. Lung tissue was collected on day 21.

### Immunofluorescence staining

Slices for antigen repair in citric acid buffer at 120°C for 20 min and 10% normal donkey serum were used to block nonspecific antigens. The antibodies used for immunofluorescence included HULMAIN A+C, AQP5, SPC, and donkey anti-rabbit LGG (H+L) highly cross-adsorbed secondary antibodies (Invitrogen, America). The primary antibody was incubated overnight at 4°C and soaked in PBS for 5 min 3 times, and the secondary antibody was incubated at room temperature for 1 h and soaked in PBS for 5 min. After staining with DAPI, the cells were soaked in PBS for 5 times. The slides were stored in the dark at 4°C and photographed with a fluorescence microscope.

### Quantitative PCR

An EZ-press RNA purification kit (B0004D, EZBioscience) was used to extract the total RNA after collecting the lung tissue. A Fast cDNA Synthesis Kit was used to reverse transcribe total RNA (#B0001, EZBio-science). The expression levels of IFN-γ and IL-17 were detected by adding IFN-γ and IL-17 primers in a LightCycler^®^ 96 Real-time Quantitative Fluorescence PCR (Roche, America) using a heat-initiated qPCR kit (TransScript^®^ Green One-Step qPCR SuperMix, AQ211-01, TransGen Biotech, Beijing, China).

### Hydroxyproline detection

An HYP assay kit (BC0255, Solarbio, China) was used to test the variation in the HYP content in the tissue samples. First, the tissue was accurately weighed. After hydrolysis, the pH was adjusted to neutral, and the supernatant was centrifuged with activated carbon. The reagent was added and then incubated in water at 60°C for 15 min. After 10 min at 3,500 rpm, the supernatant was taken for spectrophotometry at 550 nm.

### Data statistics and analysis methods

The ADT tool was used to calculate the binding free energy between compounds and receptors, and the machine learning model and related data were processed by Python version 3.6.2. Data from the biological verification experiments were statistically analyzed by GraphPad Prism 8.0 and Origin 9.0 software. The Kaplan-Meier method was used for survival curves, and data are expressed as the mean ± standard deviation. A test was used for analysis between two independent sample groups, and univariate analysis was used for comparison between multiple groups of data. *p* < 0.05 indicates that the difference is statistically significant.

## Results

### Structure-based drug design and virtual screening

AutoDock, which is open source and widely used, was selected for SBDD and virtual screening to obtain lead compounds with binding potential to the target. Previous studies have shown that drugs targeting multiple receptors may inhibit the development of IPF better than those targeting individual receptors (Cheng et al., 2019; Prieto et al., 2021). To evaluate the feasibility of screening drugs against multiple targets, we first analyzed the spatial structural similarity, sequence similarity, and physiological pathway associations of ligand-binding domains in nine receptors. The nine receptors are vascular endothelial growth factor receptor 1/2/3 (VEGFR 1/2/3), fibroblast growth factor receptor 1/2/3 (FGFR 1/2/3), platelet-derived growth factor receptor α/β (PDGF α/β), and transforming growth factor β receptor (TGF-B1R). Structural data were obtained from PDB, and for VEGFR3 and PDGFRβ lacking structural information, we used SWISS-MODEL for homology modeling (Supplementary Figures S1A,B). Furthermore, Verix-3D (Supplementary Figures S1C,D), Laplace plots (Supplementary Figures S1E,F) and PSIPRED secondary structure analysis (Supplementary Figures S1G,H) were utilized to validate the accuracy of the models, ensuring that the models we built were reasonable and effective.

**FIGURE 1:**
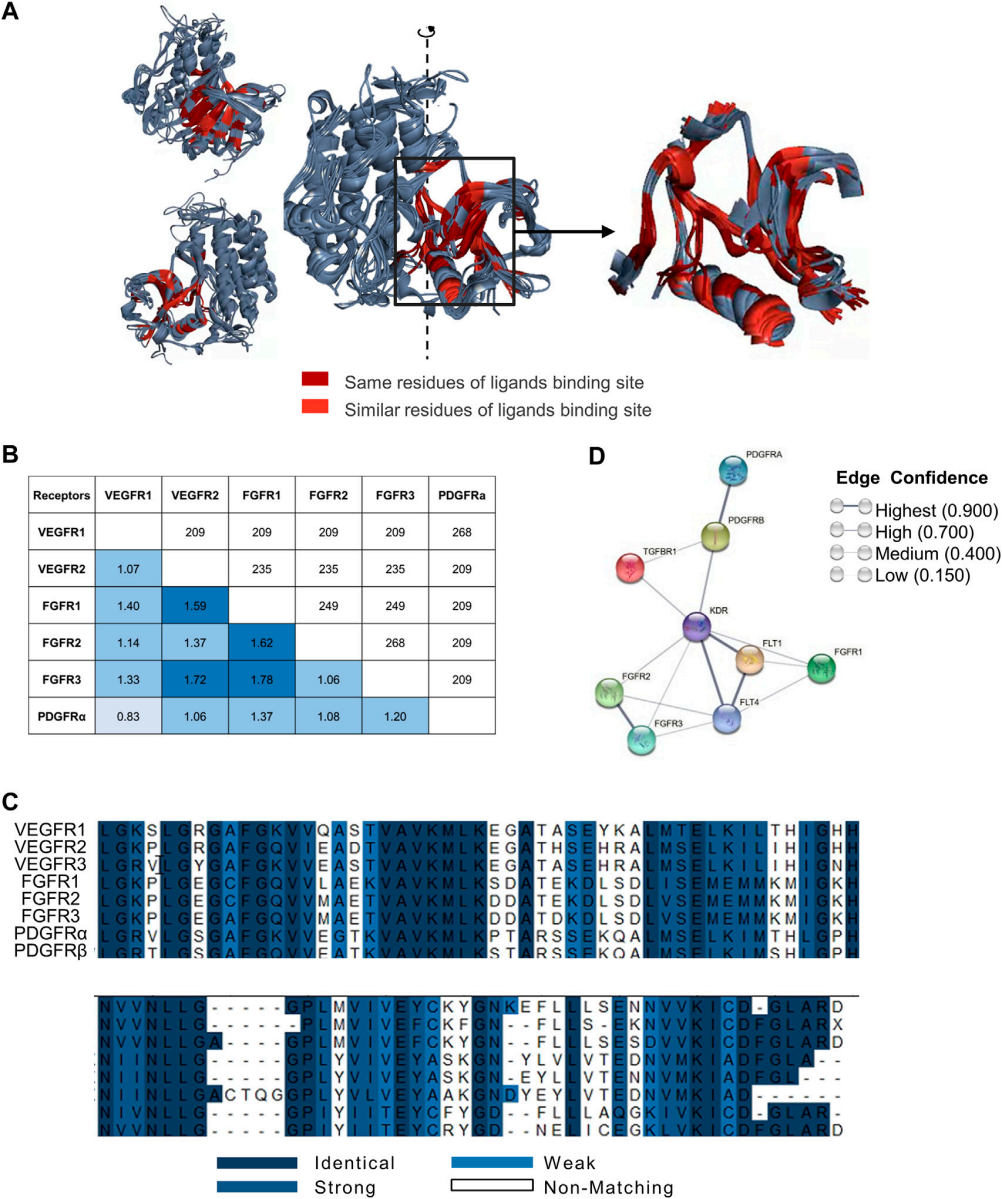
Spatial structural similarity, sequence similarity, and physiological pathway associations in nine receptors. **(A)** Superimposition of VEGFRs, FGFRs and PDGFRs. Superimposition of VEGFR1/2, FGFR1/2/3 and PDGFRα (left) and binding sites (right). The ligand-bound hydrophobic cavities from each target and superimposition of the three-dimensional structures of these cavities. We found that the residues in the cavity with the opportunity to contact the ligand are conserved, and the spatial structure is relatively the same. Dark red indicates identical targets, and bright red indicates targets with similar properties. Only the ligand binding domain similarities are marked in the figure. **(B)** Structural alignment of VEGFR1/2, FGFR1/2/3 and PDGFRα. The main-chain RMSD (angstrom) is below the diagonal, and the number of overlapping residues is above the diagonal. The main-chain RMSD of these targets was between 0.83 Å and 1.78 Å. Of the approximately 350 residues, there were between 200 and 250 residues with good overlap. This means that the spatial structures of these targets do not differ much. **(C)** Sequence alignment of VEGFR1/2/3, FGFR1/2/3 and PDGFRα/β. Blue ranges from dark to weak, indicating amino acid residues ranging from similar to unrelated. In the cavity, the proportion of the same residues was 32.3%, and the proportion of similar residues was 54.5%. This suggests that residues of the cavity are conserved. **(D)** Relevance analysis of targets from the STRING database. Nine proteins (VEGFR1: FLT1, VEGFR2: KDR, VEGFR3: FLT4, FGFR1, FGFR2, FGFR3, PDGFRα: PDGFRA, PDGFRβ: PDGFRB and TGFβR1: TGFBR1) were selected from the STRING database. Sphere points replaced several proteins, and line thickness indicates the strength of data support. Each node represents all the proteins produced by a single, protein-coding gene locus. Edges represent protein‒protein associations that are meant to be specific and meaningful, i.e., proteins jointly contribute to a shared function; this does not necessarily mean they physically bind each other. Data from the STRING database show that these targets are closely related.

To compare the structures of VEGFRs, FGFRs and PDGFRs, we selected and superimposed the three-dimensional (3D) structures of ligand-bound hydrophobic cavities from each receptor and found that the spatial structures of these targets were relatively similar (Figure 1A). Furthermore, the main-chain RMSD of these targets ranged from 0.83 Å to 1.78 Å (Figure 1B). These results suggest that the spatial structures of VEGFRs, FGFRs and PDGFRs are relatively similar.

Sequence alignment of VEGFR1/2/3, FGFR1/2/3 and PDGFRα/β revealed approximately 200–250 residues overlapping per 350 residues. Meanwhile, the proportions of the same and similar residues in hydrophobic cavities were 32.3% and 54.5%, respectively (Figure 1C). These results indicated that the residues in cavities with the opportunity to contact the ligands are conserved. The analysis of VEGFRs, FGFRs, and PDGFRs in terms of structural similarity and sequence alignment revealed that the ligand-binding domains of these receptors share a high degree of structural and sequence homology.

In addition, STRING database analysis showed that the physiological effects of VEGFRs, FGFRs, PDGFRs and TGFβ1R were closely related (Figure 1D). These analyses demonstrate that it is feasible to design broad-spectrum lead compounds that simultaneously target VEGFRs, FGFRs, PDGFRs, and TGFβ1R.

Subsequently, docking analysis and screening were performed using the processed Enamine database, as described in the methods. Candidate compounds with the highest comprehensive score that simultaneously bound the nine receptors were selected according to the ranking of the candidate binding to each target (Supplementary Figure S2).

**FIGURE 2:**
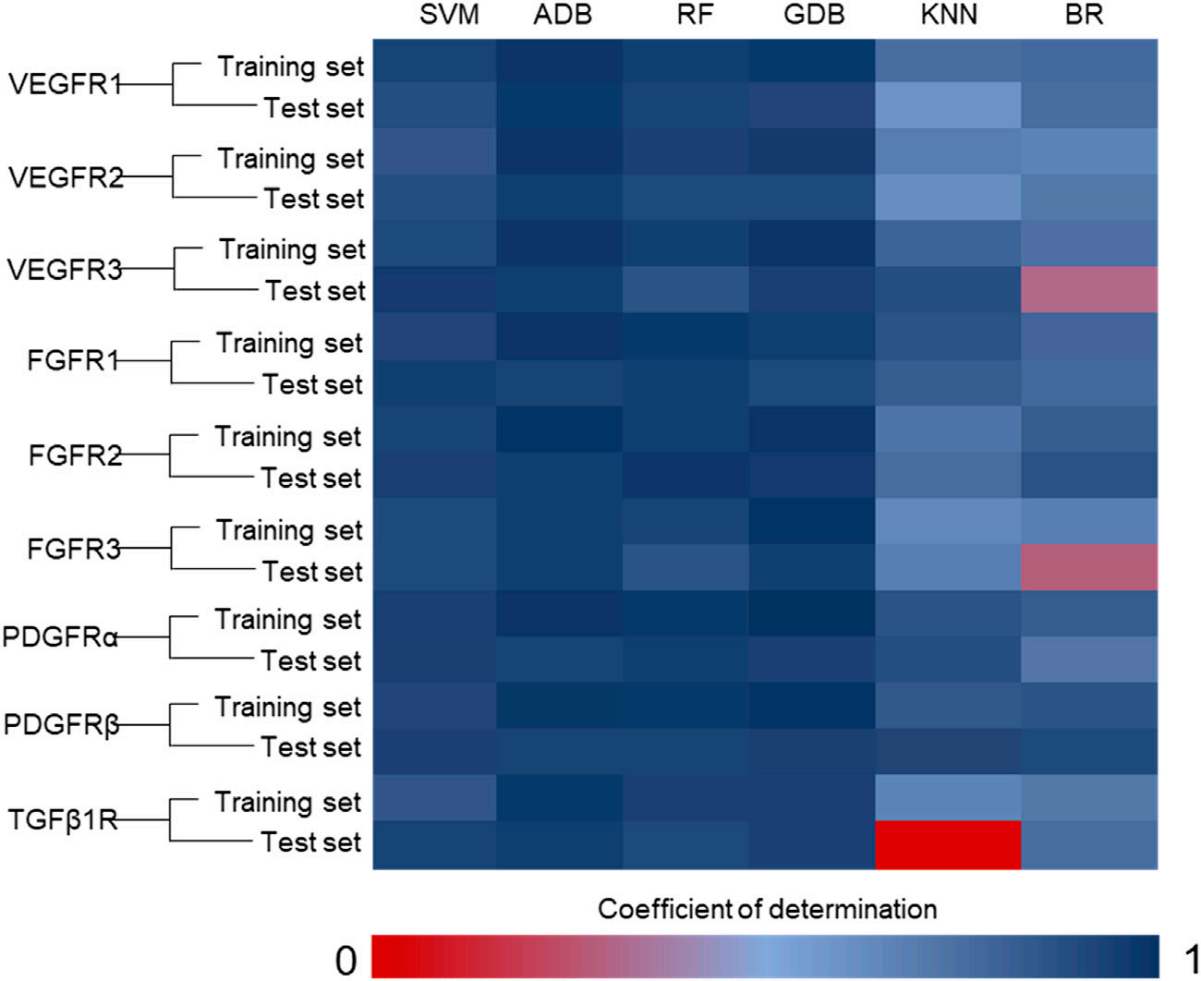
Coefficient of determination of the machine learning model. (SVM: support vector machine; ADB: AdaBoost; RF: random forest; GDB: gradient boosting; KNN: K-nearest neighbor; BR: Bayesian ridge) The integrated algorithms, such as ADB, RF and GDB, performed better than the single algorithm, and the determination coefficients of both the training set and the test set were greater than 0.85. However, due to the limitation of ligand data, the cross-validation results of these models are poor. This implies that these machine learning models are overfitting. These models will have poor accuracy in predicting unknown ligands, so we need to combine the results of virtual screening for selection. In addition, experimental data should be used to reduce the false positive rate of the model.

We then randomly assigned 80% of the ligands to the training group and 20% to the test group. Following this, the SVM, ADB, RF, GDB, KNN and BR algorithms in Scikit-Learn in Python were used for modeling, and multiple machine learning models were obtained (Figure 2). The data processing method predicted by the machine learning model was consistent with that of the virtual screening data. Consequently, 20 compounds were selected from the candidate compounds after the first round of computer screening for subsequent verification at the cellular level (Supplementary Table S1).

### High-throughput screening of antifibrotic lead compounds

The basic pathogenic change in IPF is the accumulation of collagen. During the late stage of IPF, collagen type I, which is encoded by the *COL1A2* gene, is deposited (Sgalla et al., 2018). In this study, human alveolar epithelial cells (A549) with a stable luciferase reporter system (Collagen1A2-A549) were constructed, and rapid high-throughput screening of lead compounds with transcriptional regulation of *COL1A2* was achieved. To validate that the physiologically active candidate compounds can be screened by Collagen1A2-A549 cells, we first used tyrosine kinase agonists (FGF1, TGFβ1, and PDGFα) and antagonists (NDNB, PFD, and dexamethasone) to examine the function of the engineered cells. The agonists FGF1 (73.29% increased, 10 ng/μL, 36 h, *p* < 0.05), TGFβ1 (54.25% increased, 10 ng/μL, 12 h, *p* < 0.05) and PDGFα (56.53% increased, 10 ng/μL, 36 h, *p* < 0.05), as well as the antagonists NDNB (87.24% reduction, 10 μm, 24 h, *p* < 0.05), PFD (84.72% reduction, 10 μm, 36 h, *p* < 0.05), and dexamethasone (44.95%, 50 μm, 36 h, *p* < 0.05), all worked successfully in Collagen1A2-A549 cells (Supplementary Figures S3A–F). Furthermore, we examined differences in the expression of tyrosine kinase receptors and TGFβ1R in Collagen1A2-A549 cells and human dermal fibroblasts (HDFs) by qPCR. The results showed that all selected receptors were expressed in Collagen1A2-A549 cells, especially VEGFR3, which was highly expressed (Supplementary Figure S4G). These results indicate that Collagen1A2-A549 cells can screen candidate compounds with physiological activity.

**FIGURE 3:**
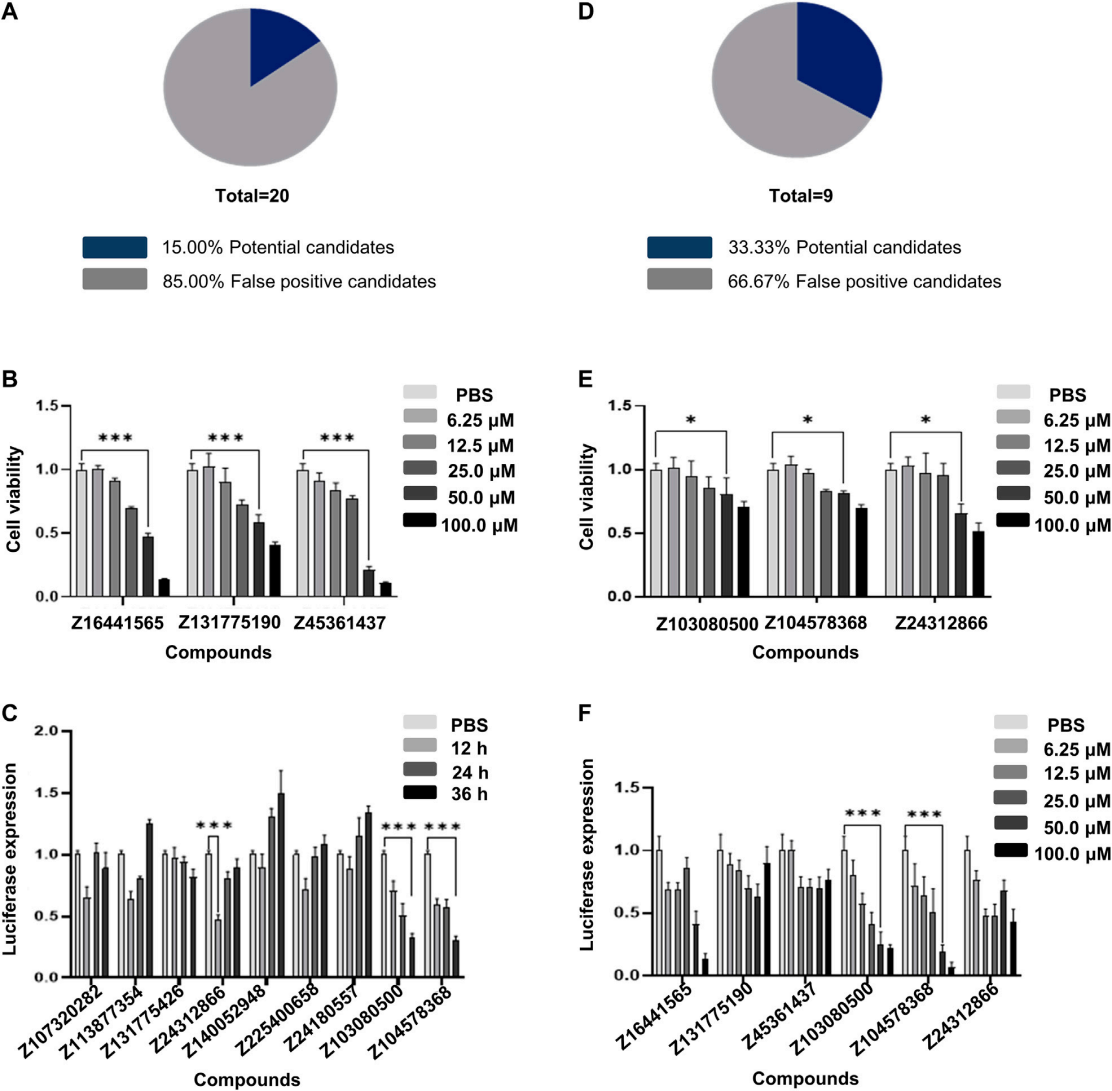
High-throughput screening using Collagen1A2-A549 cells. **(A)** False positive rate calculated with 20 compounds selected from the first round of calculation. **(B)** CCK8 cytotoxicity results of the three compounds with better performance among the 20 compounds selected in the first round. **(C)** Validation of nine compounds selected from the second round of calculation. **(D)** False positive rate calculated in the second round of calculation. **(E)** CCK8 cytotoxicity results of the three compounds with better performance among the nine compounds selected in the second round of calculation. **(F)** Validation of the concentration gradient of six compounds selected by two rounds of calculation for anti-collagen generation. All experiments were repeated three times independently.

We then empirically validated 20 of the top 100 compounds in the composite score using Collagen1A2-A549 cells. After data analysis and processing, we obtained the first round of 20 compounds, of which the false positive rate was 85% (Figure 3A). Among them, the compounds Z16441565 (58.78% inhibition rate, 50 μM, 36 h, *p* < 0.05), Z131775190 (48.70% inhibition rate, 50 μM, 36 h, *p* < 0.05) and Z45361437 (72.29% inhibition rate, 50 μM, 36 h, *p* < 0.05) effectively inhibited the transcription of collagen (Figure 3B).

To effectively reduce the false positive rate, we fed the data from the first round of compounds into the machine learning model to optimize the algorithm. After feedback learning screening, nine compounds were obtained, and the false positive rate decreased by approximately 18% compared with the first round (Supplementary Table S2), Figures 3C,D). This finding suggests that models that continuously perform machine learning to optimize screening based on results will help improve accuracy.

Through machine learning and two rounds of screening, we examined the luciferase expression of six compounds. The inhibition rates of the compounds Z103080500 and Z104578368 on *COL1A2* transcription levels were 67.88% and 69.54%, respectively, in response to 50 μmol/L treatment for 36 h (Figure 3F). Compared with that of the compounds in the first round, the cytotoxicity of the compounds Z103080500 and Z104578368 under the same conditions was 29.84% and 29.93%, respectively, indicating that these two compounds have lower cytotoxicity to A549 cells (Figure 3E). The results proved that these two compounds, Z103080500 and Z104578368, have antifibrotic activity at the cellular level. Therefore, we then characterized these two compounds and evaluated their activities in animals.

### Characterization of Z103080500 and Z104578368

**FIGURE 4:**
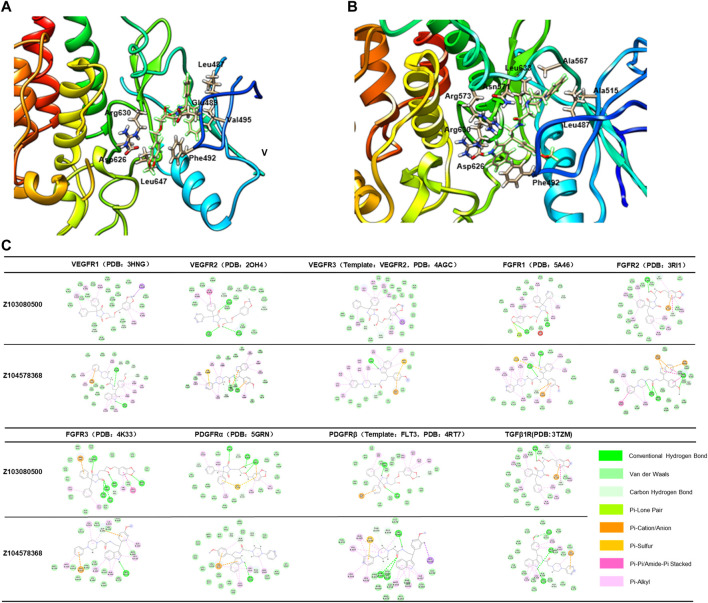
Binding of compounds Z03080500 and Z104578368 to receptors. **(A)** Binding mode of Z103080500 with FGFR2. **(B)** Binding mode of Z104578368 with FGFR2. **(C)** Binding mode of ligands (Z103080500 and Z104578368) and their targets.

According to the results of ADT analysis, both Z103080500 and Z104578368 have good combination potential with the receptors (Figure 4). Taking FGFR2 (PDB: 3RI1) as an example, Z103080500 and Z104578368 were well locked in a hydrophobic cavity. Z103080500 (Figure 4A) and Z104578368 (Figure 4B) form a close binding force with the residues in the hydrophobic cavity. The methods of combining Z103080500 and Z104578368 with other targets are shown in Figure 4C.

According to the results of machine learning model scoring, Z103080500 and Z104578368 also have good biological activity *in vivo* (Supplementary Table S3). Compared to NDNB, Z103080500 and Z104578368 can simultaneously bind TGFβ1R, block the Smad pathway, and reduce the secretion of collagen, thus inhibiting the occurrence of pulmonary fibrosis. Compared to PFD, Z103080500 and Z104578368 could simultaneously bind VEGFRS, FGFRS and PDGFRS and inhibit the effects of various fibrotic factors. Therefore, Z103080500 and Z104578368 have a broader affinity for anti-pulmonary fibrosis targets than NDNB and PFD. However, the specific combination mode and mechanism remain to be further confirmed.

### Z103080500 and Z104578368 can inhibit the early inflammatory response and delay pulmonary fibrosis

**FIGURE 5:**
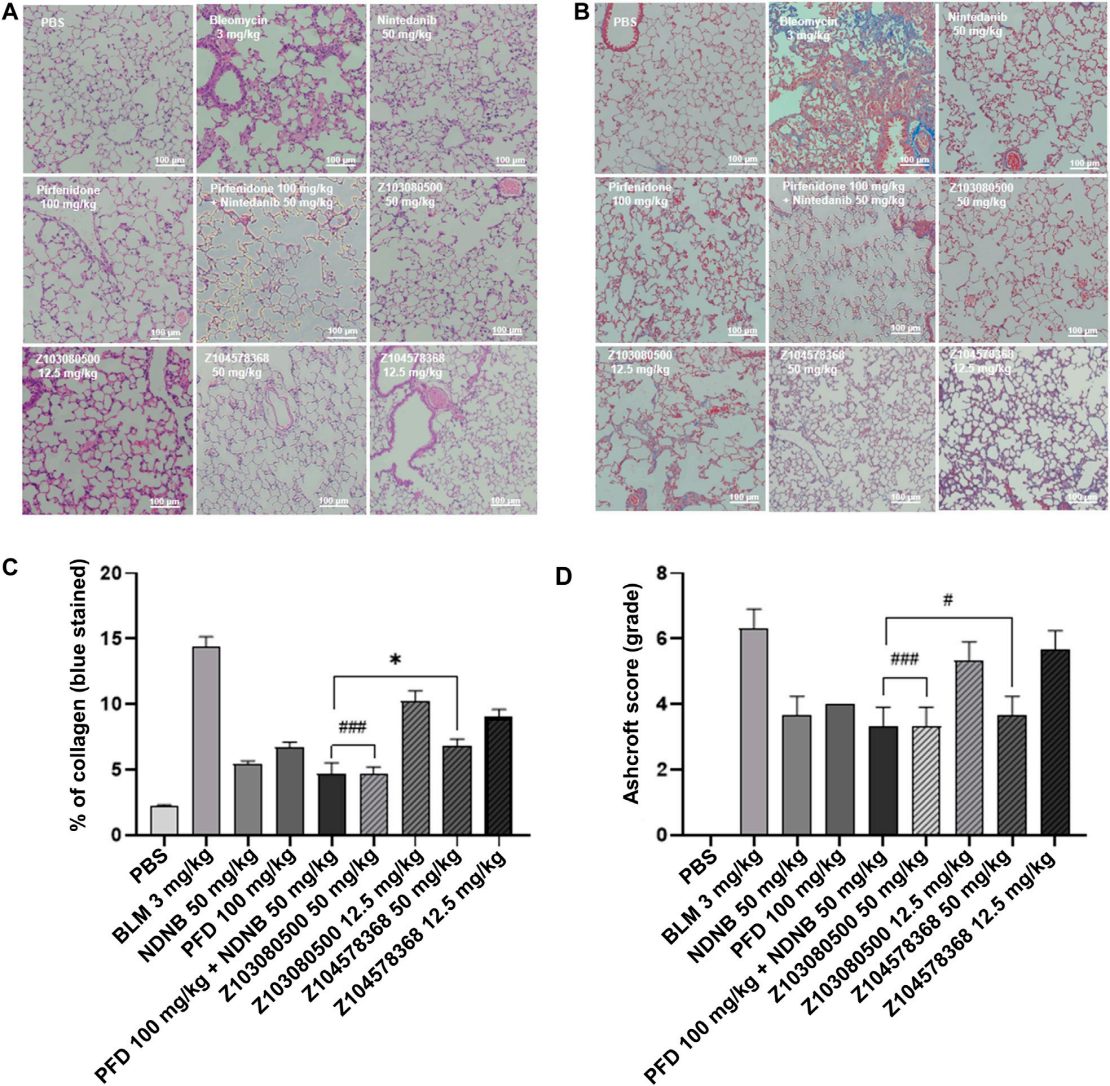
HE-stained sections and Masson trichrome-stained sections. **(A)** HE-stained section. **(B)** Masson trichrome-stained section. Grouping is marked in the diagram. **(C)** Quantitative analysis of Masson-stained sections. **(D)** Ashcroft score. Doses and groups have been marked on the abscissa. All experiments were performed in triplicate independently. BLM: BLM. NDNB: NDNB. PFD: PFD.

We constructed a BLM-induced pulmonary fibrosis model in C57BL/6 mice to evaluate the antifibrotic effect of the lead compounds Z103080500 and Z104578368 *in vivo*. According to the HE staining results, inflammatory cells in the groups treated with high concentrations of the compounds Z103080500 and Z104578368 were significantly reduced. Moreover, the effects of Z103080500 and Z104578368 were dose dependent. The effect in the Z103080500 and Z104578368 high-concentration groups was better than that in the low-concentration groups and better than those of the NDNB plus PFD combined group (Figure 5A).

The results of Masson staining showed that compounds Z103080500 (67.15% reduction, *p* < 0.05) and Z104578368 (52.72% reduction, *p* < 0.05) in the high-concentration group induced significant decreases in collagen levels (Figure 5B). Furthermore, Masson staining slices of the Z103080500 and Z104578368 high-concentration groups showed that the lung tissue structure was relatively normal, there were no significant structural changes, but collagen was secreted (Figure 5B). Quantitative analysis of the Masson-stained sections showed that the collagen levels in the high-concentration Z104578368 group were slightly higher than those in the NDNB plus PFD combined group (*p* < 0.05) and significantly lower than those in the model group (*p* < 0.05), while the collagen levels in the high-concentration Z103080500 group were equivalent to those in the NDNB plus PFD combined group (Figure 5C). The Ashcroft score was consistent with these findings (Figure 5D). The HE and Masson staining results suggested that the compounds Z103080500 and Z104578368 could effectively delay inflammation in lung tissue.

**FIGURE 6:**
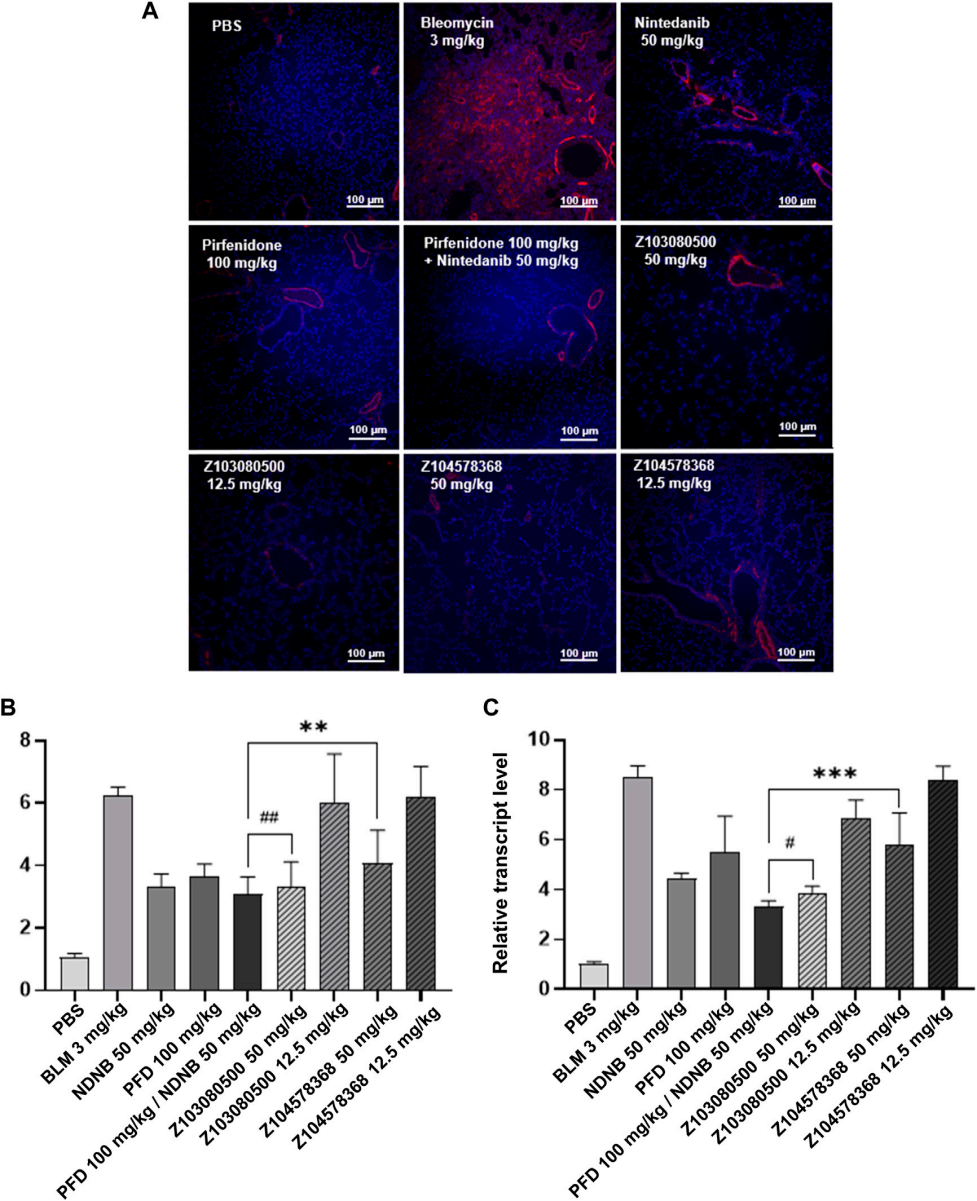
Immunofluorescent staining and relative transcript levels of inflammatory factors. **(A)** α-SMA immunofluorescently stained section. Grouping and doses are marked in the diagram. α-SMA, red. DAPI, blue. **(B)** Relative transcript level of IFN-γ. **(C)** Relative transcript level of IL-17. Doses and groups have been marked on the abscissa. All experiments were performed in triplicate independently. α-SMA, Alpha-smooth muscle actin; BLM, BLM; NDNB, NDNB; PFD, PFD.

In addition, relevant markers of pulmonary fibrosis were detected. Z103080500 and Z104578368 decreased the expression of α-SMA in both the high and low concentration groups. This finding indicates that the compounds Z103080500 and Z104578368 can effectively inhibit fibrosis (Figure 6A). Furthermore, the results showed that high concentrations of Z103080500 and Z104578368 could effectively reduce the expression of IFN-γ and IL-17. Specifically, the effect of Z103080500 in the high concentration group decreased the expression of IFN-γ (Figure 6B) and IL-17 (Figure 6C) by 54.87% and 47.01%, respectively, which was better than the effects of NDNB plus PFD combination group. However, the high concentration Z104578368 treatment was slightly worse than the NDNB plus PFD combined group, decreasing the expression of IFN-γ (Figure 6B) and IL-17 (Figure 6C) by 37.29% and 49.38%, respectively.

**FIGURE 7:**
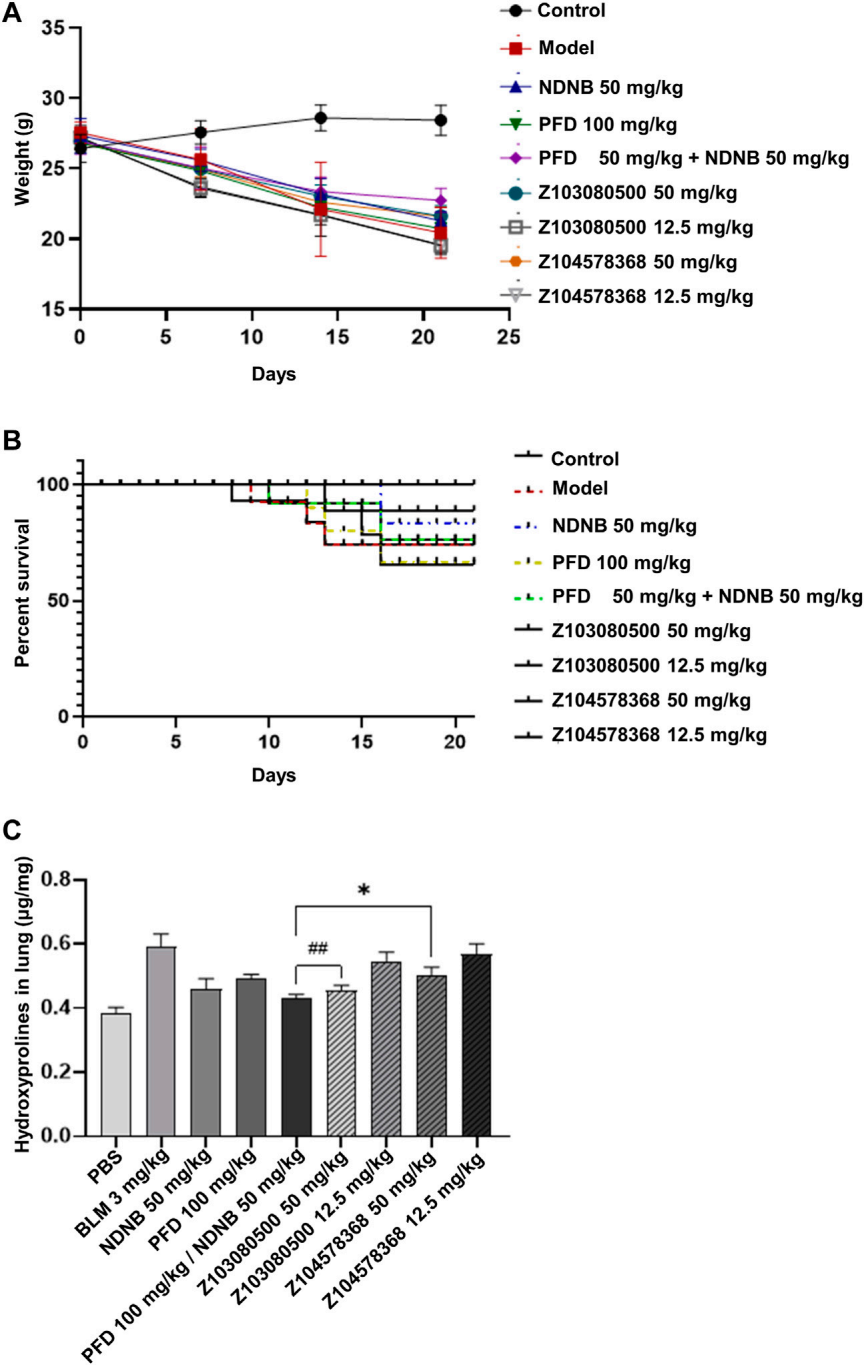
Body weight change, survival, and biomarker expression. Changes in body weight **(A)** and survival **(B)** of BLM mice throughout the experiment. **(C)** The expression of Hyp in BLM mice (N = 6-7 each group). Doses and groups have been marked on the abscissa. All experiments were performed in triplicate independently. BLM: BLM. NDNB: NDNB. PFD: PFD. Hyp: Hydroxyproline.

The body weights of the mice in the model group, positive control group and experimental group decreased to different degrees. There was no significant difference in weight loss among the three groups, and the mice in the control group gained weight steadily (Figure 7A). In terms of mortality, the model group, PFD group and low-concentration PFD groups had higher mortality rates of 30%. The NDNB group and high-concentration Z103080500 group had the lowest mortality rates (10%) (Figure 7B). This finding suggests that NDNB and a high concentration of Z103080500 may improve the survival of IPF mice.

HYP content analysis was also performed. The data showed that high concentrations of Z103080500 and Z104578368 could effectively reduce the expression of HYP. Z103080500 reduced the expression of HYP by 23.15%, which was better than the effect of NDNB plus PFD combination treatment. In contrast, the effect of Z104578368 was slightly worse than that in the NDNB plus PFD combination group, and the expression of HYP was reduced by 15.24% (Figure 7C). These results demonstrate the anti-IPF efficacy of these two lead compounds, especially Z103080500.

## Discussion

Over the last few decades, computer-aided drug design has emerged as a powerful technique playing a crucial role in the development of new drug molecules (Kaur and Khatik, 2021). It greatly speeds up new drug design, saving manpower and material resources for creating new drugs. Computer-aided quantification was used for pulmonary fibrosis in patients with lung cancer (Iwasawa et al., 2019). Based on the structure and properties of receptors, SBDD looks for ligand molecules that can specifically bind to it, in which virtual screening is an important method. Considering the similarity of the nine protein functional groups involved in fibroblast activation, including VEGFRs, FGFRs, PDGFRs, and TGF-β1R, virtual screening methods can be used to select small molecular compounds that have effects on these receptors. Since virtual screening is based on the target-ligand binding relationship, it is impossible to accurately predict ligand activity *in vivo* (Mouchlis et al., 2021).

Through SBDD, virtual screening, and machine learning, we ended up with two lead compounds, Z103080500 and Z104578368. These two compounds have broader affinities for antifibrotic targets than NDNB and PFD. Compared to NDNB, Z103080500 and Z104578368 could simultaneously bind to TGFβ1R (Figure 4), reduce the secretion of collagen (Figures 6, 7), and inhibit the occurrence of pulmonary fibrosis by blocking the Smad pathway (Richeldi et al., 2014). Compared to PFD, Z103080500 and Z104578368 could simultaneously bind VEGFRs, FGFRs and PDGFRs (Figure 4) and inhibit the effects of various fibrotic factors (Lederer and Martinez, 2018). However, the specific binding mode and mechanism remain to be further confirmed. It is worth noting that the single virtual screening high scores and machine learning prediction high scores did not ensure compound activity, and even compounds with high combined scores may have the possibility of false positives.

Data feedback optimization could improve our screening efficiency, and useless data play a large role. After feedback optimization of the first round of screening data from collagen1A2-A549 cells, the false positive rate of the machine learning model was reduced from 75% to 66%. Advanced algorithms such as machine learning models are increasingly being used in drug discovery (Elbadawi et al., 2021). Machine learning techniques improve decision-making in pharmaceutical data across various applications, such as QSAR analysis, hit discoveries, and *de novo* drug architectures, to retrieve accurate outcomes (Carracedo-Reboredo et al., 2021). Compared with virtual screening, machine learning is not dependent on the accuracy of the target model and can effectively predict ligand activity. However, the lack of data is one of the factors limiting its use, so it is particularly important to continuously optimize the screening and prediction model with experimental data (Carpenter and Huang, 2018). In drug discovery, researchers tend to discard data on poorly performing compounds, but these data often tell computational models which compounds to discard. “Dusky” data can be useful (Patel et al., 2020).

Collagen1A2-A549 cells could effectively improve screening efficiency. After preliminary screening of collagen1A2-A549 cells, both Z103080500 and Z104578368 showed good performance in animal models (Figures 6, 7). Furthermore, these experiments demonstrated that targets, agonists, and inhibitors related to the expression of collagen1A2 in A549 cells exhibited corresponding activity. However, Z103080500 and Z104578368 showed strong toxicity to collagen1A2-A549 cells, which needs to be optimized and improved in future research. The construction of similar screening cells using HDFs has been reported (Hoeck and Woisetschläger, 2001; Zhao et al., 2019). However, the state of HDFs is easily affected by the environment, and the number of passages is limited. Therefore, A549 cells are more universal than HDFs. Previous studies have shown that type III collagen is dominant in the early stage of pulmonary fibrosis and that type I collagen is dominant in the late stage (Snijder et al., 2019). When fibroblasts are damaged by chemical or physical factors, they secrete collagen to repair the interstitial tissues of the lung, resulting in pulmonary fibrosis. At present, the evaluation of the progression of pulmonary fibrosis is mainly performed by monitoring changes in important pulmonary fibrosis indicators before and after the administration of drugs (Thannickal et al., 2004; Lederer and Martinez, 2018; Sgalla et al., 2018). The commonly used monitoring methods are Western blotting and ELISA. However, these methods are susceptible to many factors, such as the cell state, the environment, and experimental methods, and cannot accurately and temporally evaluate the dynamic changes in fibrosis indicators before and after drug administration.

Z103080500 and Z104578368 showed antifibrotic potential at the cellular and animal levels. Z103080500 and Z104578368 showed strong antifibrotic effects at the cellular level, but both also showed strong cytotoxicity. The performance of Z103080500 and Z104578368 in animal models was better than that of PFD or NDNB and similar to that of PFD plus NDNB. Z103080500 and Z104578368 require lower doses, which means that Z103080500 and Z104578368 have the potential to be lead compounds for inhibiting IPF. However, the solubility of Z103080500 and Z104578368 in PBS or water is low, suggesting that the bioavailability of Z103080500 and Z104578368 may be low. Furthermore, it may be difficult for us to make appropriate dosing forms of Z103080500 and Z104578368 for drug administration. This difficulty may limit the medicinal properties of these compounds.

Recently, prodrug strategies have been utilized to promote physicochemical, biopharmaceutical and pharmacokinetic properties such as permeability, solubility, bioavailability, chemical stability and metabolism of molecules presenting poor drug-like properties (Stella and Nti-Addae, 2007; Sanches and Ferreira, 2019). For example, placing a polar functional group in the structure of a molecule with limited aqueous solubility should enhance the water solubility of these two compounds. Some commercially approved have been regenerated based on this idea. Another excellent example of improving oral delivery or bioavailability is placing a nonionizable functionality in the structure, such as sulfoxide groups. Due to the higher polarity of sulfoxide groups, the sulfide metabolites of drugs can better interact with solvents and can be used as a water-soluble drug delivery strategy.

In conclusion, both Z103080500 and Z104578368 show good potential for inhibiting IPF in cell and animal models. We will further confirm the pharmacological mechanism of both compounds and reduce the toxicity of these compounds by optimizing their structures and improving their medicinal properties.

## Data Availability

All the data obtained from the present study are available from the corresponding author upon reasonable request.
