# Supplementary material for: Virtual screening and activity evaluation of multitargeting inhibitors for idiopathic pulmonary fibrosis
Source: Front Pharmacol. 2022 Sep 8;13:998245. doi: 10.3389/fphar.2022.998245 (PMC9493029; doi:10.3389/fphar.2022.998245)
Supplement: Supplementary file 1 [file DataSheet1.PDF]

Virtual screening and activity evaluation of multitargeting inhibitors for idiopathic pulmonary fibrosis

Rui Wang [1,†], Jian Xu [2,†], Rong Yan [1, †], Huanbin Liu [2,†], Jingxin Zhao [2], Yuan Xie [2], Wenbin Deng [2], Weiping Liao [3,*], Yichu Nie[1,2,*]

1. Clinical Research Institute, The First People's Hospital of Foshan, Foshan, 528000, China.

2. School of Pharmaceutical Sciences (Shenzhen), Shenzhen campus of Sun Yat-sen University, Shenzhen, 518107, China.

3. Foshan Fourth People's Hospital, Foshan, 528000, PR China.

[†] These authors contributed equally to this work.

Corresponding authors:

* Dr. Yichu Nie, The First People's Hospital of Foshan & Sun Yat-sen University Foshan Hospital, Foshan, 528000, PR China. Email: nieyichu2@126.com

* Dr. Weiping Liao, Foshan Fourth People's Hospital, Foshan, 528000, PR China. Email: fsliaoweiping@21cn.com

.

Supporting Information

Supplementary Tables

Supplementary table 1. Consensus score of the first round of calculation

Compounds	Caculation	VEGFR1	VEGFR2	VEGFR3	FGFR1	FGFR2	FGFR3	PDGFRα	PDGFRβ	TGFβ1R	Total
Z24312866	Binding energy	1	2	3	2	1	2	1	2	2	16
	-lg(IC50)	2.45	2.41	2.49	2.32	2.20	1.36	1.21	1.79	2.51	19
Z2471178282	Binding energy	2	1	1	2	2	1	2	2	1	16
	-lg(IC50)	2.51	1.21	2.77	2.52	1.26	1.58	1.76	1.40	1.42	16
Z2527705486	Binding energy	2	2	3	3	2	2	2	3	1	19
	-lg(IC50)	1.11	2.05	2.30	1.98	1.19	2.35	1.77	1.84	2.11	17
Z295866094	Binding energy	2	3	2	1	3	2	2	3	1	18
	-lg(IC50)	1.64	2.27	2.67	1.77	1.19	2.58	1.65	1.59	2.31	18
Z46489623	Binding energy	3	2	2	2	2	1	1	1	2	17
	-lg(IC50)	2.42	2.14	1.44	2.60	2.25	1.39	2.34	1.68	2.42	19
Z51225855	Binding energy	3	2	2	3	1	3	1	2	2	19
	-lg(IC50)	2.62	1.24	1.71	1.26	2.24	2.34	1.60	2.07	1.72	17
Z56819784	Binding energy	1	3	1	2	2	2	2	2	3	18
	-lg(IC50)	2.10	2.51	2.69	2.58	1.62	1.20	1.95	1.95	2.22	19
Z14003605	Binding energy	1	2	2	2	2	1	2	3	1	17
	-lg(IC50)	2.25	2.30	2.73	1.87	1.50	2.52	2.32	1.86	2.26	20
Z16441565	Binding energy	1	3	2	1	1	1	1	2	2	16
	-lg(IC50)	2.26	1.27	1.21	1.12	2.69	1.46	2.65	2.63	2.68	18
Z19204438	Binding energy	2	2	2	1	2	1	2	1	1	16
	-lg(IC50)	2.66	2.20	2.29	2.62	2.08	1.99	1.70	1.26	1.41	18
Z225110050	Binding energy	3	2	1	1	2	2	2	2	2	17
	-lg(IC50)	2.45	1.26	1.38	2.22	1.27	2.64	1.38	2.16	1.82	17
Z226269158	Binding energy	1	2	2	2	2	2	2	1	1	16
	-lg(IC50)	2.52	2.36	1.50	2.60	2.22	1.38	2.58	1.83	1.25	18
Z2471178242	Binding energy	2	3	1	1	1	3	2	1	2	17
	-lg(IC50)	1.32	2.06	2.63	1.80	2.54	2.59	2.21	2.75	2.46	20
Z65821478	Binding energy	2	2	1	1	2	2	1	1	3	15
	-lg(IC50)	1.46	2.03	1.12	2.29	2.03	1.57	2.70	1.28	1.35	16
Z98224484	Binding energy	3	2	2	1	2	2	2	2	1	18
	-lg(IC50)	2.11	2.72	2.22	1.74	2.04	1.38	1.94	1.97	1.69	18
Z44584637	Binding energy	3	3	1	2	2	2	2	2	2	19
	-lg(IC50)	1.14	2.21	2.55	2.68	1.71	1.46	1.36	1.50	2.18	17
Z126244654	Binding energy	2	2	2	1	2	2	2	2	2	16
	-lg(IC50)	1.28	2.36	2.52	2.66	2.35	1.99	2.38	1.87	1.53	19
Z131775190	Binding energy	2	3	2	2	2	2	1	2	2	18
	-lg(IC50)	1.42	1.94	2.19	2.73	2.40	2.43	1.40	2.70	2.65	20
Z45361437	Binding energy	1	2	2	1	2	3	2	2	1	17
	-lg(IC50)	2.70	1.23	2.12	2.03	1.22	2.48	2.59	1.88	2.70	19
Z51206769	Binding energy	2	2	1	2	1	2	2	2	2	15
	-lg(IC50)	1.70	2.34	2.70	1.62	2.50	1.99	2.63	1.99	2.01	19
Z25756242	Binding energy	2	2	2	1	1	1	2	2	1	15
	-lg(IC50)	1.65	1.96	2.26	2.38	2.31	2.72	1.67	1.73	2.51	19

Supplementary table 2. Prediction of virtual screening and machine learning

Compounds	Caculation	VEGFR1	VEGFR2	VEGFR3	FGFR1	FGFR2	FGFR3	PDGFRα	PDGFRβ	TGFβ1R	Total
Z103080500	Binding energy	1	1	2	2	2	2	2	3	1	16
	-lg(IC50)	1.85	1.85	1.62	1.41	2.34	1.32	2.11	1.70	1.80	16
Z104578368	Binding energy	3	3	3	2	2	2	1	1	3	18
	-lg(IC50)	2.18	2.18	1.25	2.50	1.56	2.40	2.31	1.57	1.50	17
Z107320282	Binding energy	3	3	2	2	2	3	2	2	2	20
	-lg(IC50)	2.04	2.04	2.01	2.11	1.94	1.40	2.38	1.67	2.59	18
Z113877354	Binding energy	1	1	1	3	2	3	3	2	2	17
	-lg(IC50)	1.71	1.71	2.18	2.42	2.64	1.71	1.34	1.75	2.01	17
Z131775426	Binding energy	1	1	2	2	2	1	2	2	2	15
	-lg(IC50)	1.34	1.34	2.37	2.17	1.55	2.69	2.77	1.46	1.31	17
Z140052948	Binding energy	3	3	2	2	1	3	2	2	1	19
	-lg(IC50)	2.59	2.59	1.36	1.58	2.24	2.32	1.63	1.94	1.12	17
Z225400658	Binding energy	1	1	1	2	2	2	2	1	2	16
	-lg(IC50)	2.14	2.14	1.55	1.83	1.72	1.60	1.49	1.78	1.63	16
Z24180557	Binding energy	3	3	2	3	3	2	2	3	2	22
	-lg(IC50)	2.37	2.37	2.48	1.27	2.06	1.32	1.57	1.76	1.96	17

Supplementary table 3. Consensus score of the second round of calculation

Targets	Tools	Calculation method	Nintedanib	Pirfenidone	Z103080500	Z104578368
VEGFR1	Autodock	Binding energy(Kcal/mol)	-8.43	/	-8.45	-9.15
		Inhibition constants(nM)	649.29	/	627.72	191.70
	Machine Learning	-lg(IC50)	7.47	/	8.12	7.12
VEGFR2	Autodock	Binding energy(Kcal/mol)	-9.15	/	-9.98	-8.49
		Inhibition constants(nM)	191.70	/	47.16	591.67
	Machine Learning	-lg(IC50)	7.89	/	7.48	8.32
VEGFR3	Autodock	Binding energy(Kcal/mol)	-9.30	/	-10.23	-7.98
		Inhibition constants(nM)	150.55	/	31.01	1384.18
	Machine Learning	-lg(IC50)	7.89	/	9.12	6.48
FGFR1	Autodock	Binding energy(Kcal/mol)	-10.26	/	-9.84	-9.95
		Inhibition constants(nM)	29.38	/	59.95	49.86
	Machine Learning	-lg(IC50)	7.16	/	9.65	8.16
FGFR2	Autodock	Binding energy(Kcal/mol)	-8.45	/	-8.99	-8.46
		Inhibition constants(nM)	628.78	/	254.20	623.49
	Machine Learning	-lg(IC50)	7.43	/	8.45	7.12
FGFR3	Autodock	Binding energy(Kcal/mol)	-9.01	/	-9.99	-9.30
		Inhibition constants(nM)	243.68	/	46.92	149.54
	Machine Learning	-lg(IC50)	6.97	/	7.46	8.48
PDGFRα	Autodock	Binding energy(Kcal/mol)	-9.46	/	-9.85	-9.32
		Inhibition constants(nM)	115.08	/	59.64	144.57
	Machine Learning	-lg(IC50)	7.23	/	8.45	7.41
PDGFRβ	Autodock	Binding energy(Kcal/mol)	-8.45	/	-9.65	-9.49
		Inhibition constants(nM)	626.66	/	83.20	109.21
	Machine Learning	-lg(IC50)	7.19	/	8.15	9.12
TGFβ1R	Autodock	Binding energy(Kcal/mol)	/	-8.85	-9.32	-9.36
		Inhibition constants(nM)	/	323.13	144.57	136.04
	Machine Learning	-lg(IC50)	/	6.49	8.46	8.15

Supplementary Figures

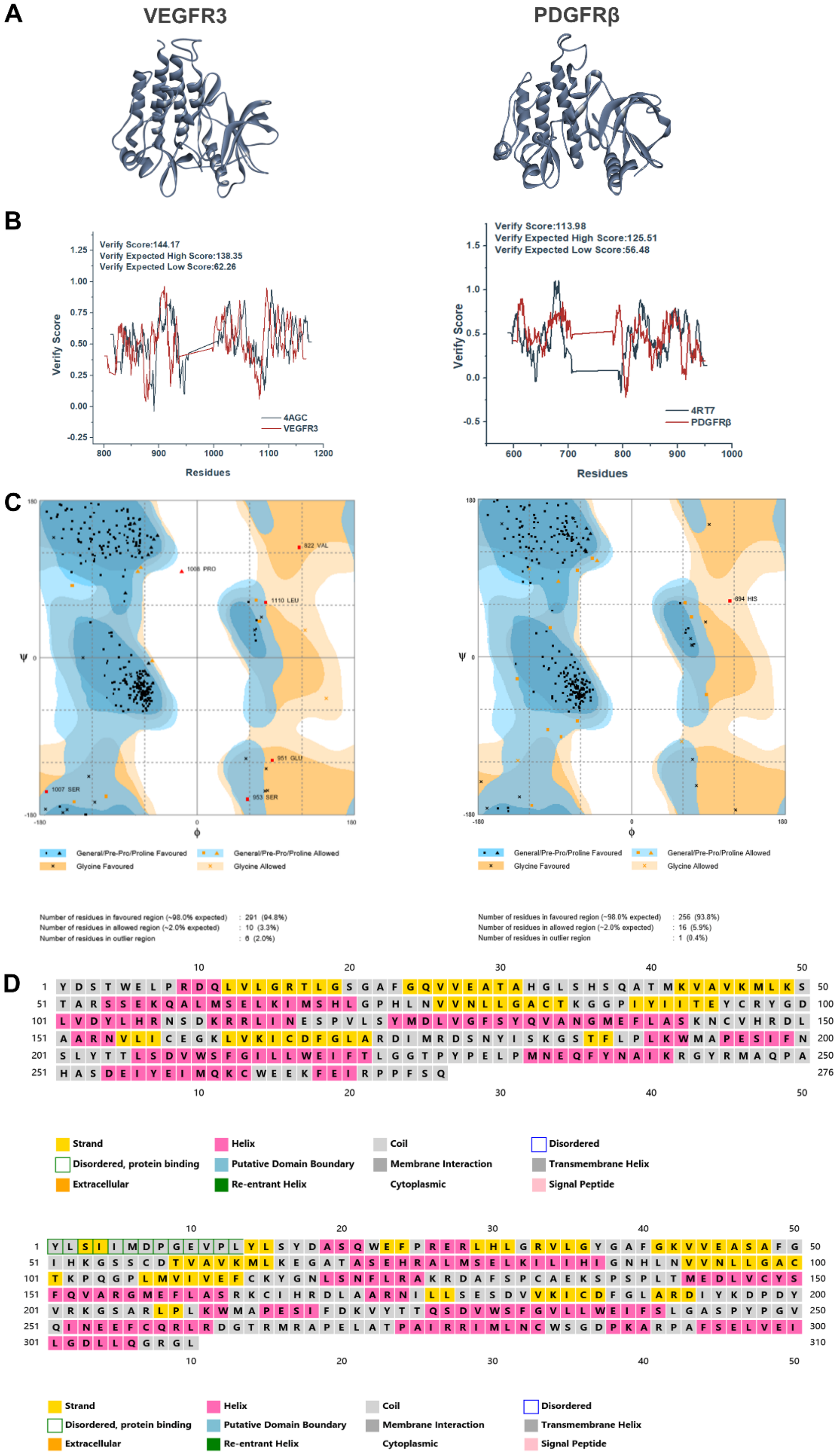

Supplementary figure 1. Homology modeling and structural validation of VEGFR3 and PDGFRβ. (A) Homology Modelling of VEGFR3 and PDGFRβ Protein sequences of VEGFR3 (Identifier: p17948-1) and PDGFRβ (Identifier: p16234-1) were obtained from Uniprot database. We used SWISS-MODEL to complete the homology modeling of VEGFR3 (Template：VEGFR2，PDB：4AGC) and PDGFRβ (Template：FLT3，PDB：4RT7). (B) Verify 3D of VEGFR3 and PDGFRβ. We used SAVESv6.0 to complete the verify 3D of VEGFR3 and PDGFRβ. Total score of VEGFR3 is 144.17, which is higher than expected high score. Total score of PDGFRβ is 113.98, which is higher than expected low score and close to expected high score. Thus, structure of VEGFR3 and PDGFRβ are reasonable and reliable. (C) Ramachandran maps of VEGFR3 and PDGFRβ. The proportion of residues of VEGFR3 in favoured region is 94.8% and only 2.0% residues are in outlier region. Similarly, the proportion of residues of PDGFRβ in favoured region is 93.8% and only 0.4% residues are in outlier region. (D) Secondary structure prediction of VEGFR3 and PDGFRβ. According to the prediction of PSIPRED, there are no disordered residues in VEGFR3 and PDGFRβ. At the same time, their secondary structure is basically the same as the template.

A

$$Consensus\ score = \left(\sum_{Targets}^{n} S_{Targets} \right) \Big/ n$$

$Targets$: Reference to the targets in this study.

$S_{Targets}$: Rank scores of candidates in each independent calculation.

n: Quantity of calculation. This is for calculating the arithmetic mean of each independent calculation.

B

Rank	Score
Top 5%	4
Top 10%	3
Top 20%	2
Top 30%	1
Top 50%	0
After 30%	-1
After 20%	-2
After 10%	-3
After 5%	-4

Supplementary figure 2. Calculation and standard of consensus score. (A) The calculation method of consensus score. $STargets$ are given according to the standard. A single virtual screening of a single target is considered an independent calculation. The prediction of a single machine learning model for a single target is treated as an independent calculation. (B) The standard of consensus score. Scores of Virtual screening and machine learning were ranked from highest to lowest.

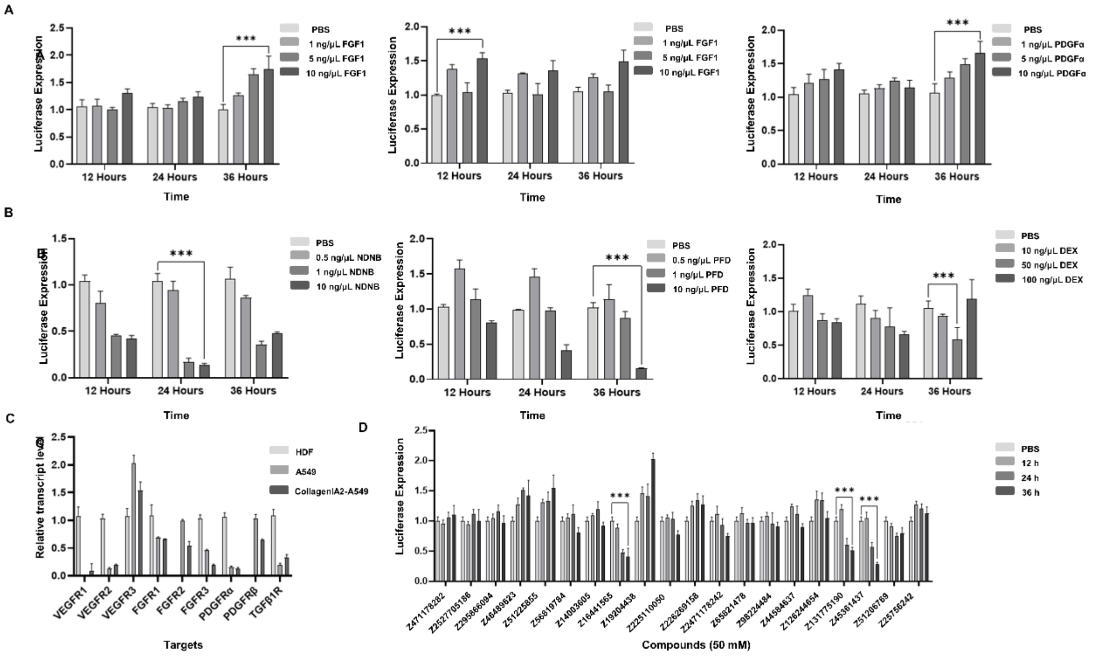

Supplementary figure 3. Functional validation of collagenIA2-A549. (A) Validation of FGF1(left), TGF-β1(middle) and PDGFRα (right). (B)Validation of Nintedanib (left), pirfenidone (middle) and dexamethasone (right). (C) Expression of VEGFR1/2/3, FGFR1/2/3, and PDGFRα/β in COL1A2-luciferase A549 cells. (D) Validation results of 20 compounds selected from the first round of calculation.
